# Partial Substitution of Organic Fertilizer with Chemical Fertilizer Improves Soil Biochemical Attributes, Rice Yields and Restores Bacterial Community Diversity in a Paddy Field

**DOI:** 10.3389/fpls.2022.895230

**Published:** 2022-06-02

**Authors:** Anas Iqbal, Liang He, Izhar Ali, Pengli Yuan, Abdullah Khan, Zhang Hua, Shanqing Wei, Ligeng Jiang

**Affiliations:** ^1^College of Life Science and Technology, Guangxi University, Nanning, China; ^2^College of Agriculture, Guangxi University, Nanning, China

**Keywords:** paddy field, organic manure, chemical fertilizer, soil chemical properties, bacterial community, grain yield

## Abstract

Conventional farming systems are highly reliant on chemical fertilizers (CFs), which adversely affect soil quality, crop production and the environment. One of the major current challenges of current agriculture is finding ways to increase soil health and crop yield sustainably. Manure application as a substitute for CF is an alternative fertilization strategy for maintaining soil health and biodiversity. However, little is known about the complex response of soil bacterial communities and soil nutrients to manure and CFs application. This study reports the response of soil nutrients, rice yield, and soil microbial community structure to 2 years of continuous manure and CFs application. The study consisted of six treatments: no N fertilizer control (Neg-Con); 100% CF (Pos-Con); 60% cattle manure (CM) + 40% CF (High-CM); 30% CM + 70% CF (Low-CM); 60% poultry manure (PM) + 40% CF (High-PM), and 30% PM + 70% CF (Low-PM). We used high-throughput sequencing of 16S ribosomal RNA gene amplicons to characterize the soil bacterial communities. Results revealed that the addition of manure significantly altered the soil bacterial community composition and structure; and enhanced the relative abundance of phyla Proteobacteria, Chloroflexi, Firmicutes, Acidobacteria, and Planctomycetes. Organic fertilizer treatments, particularly high CM and PM had the highest measured soil bacterial diversity of all treatments. Similarly, integrated application of manure and CFs increased the soil biochemical traits [i.e., pH, total N (TN), soil organic C (SOC), microbial biomass N (MBN), and microbial biomass C (MBC)] and rice grain yield. Average increases in SOC, TN, MBN, and MBC were 43.66, 31.57, 24.34, and 49.45%, respectively, over the years in the High-PM compared with Pos-Con. Redundancy analysis showed that the dominant bacteria phyla were correlated with soil pH, SOC, TN, and microbial biomass, but the relative abundance of Proteobacteria was strongly correlated with environmental factors such as soil pH, SOC, TN, and MBC. We employed a structural equation model to examine the relationship between microbial biomass, soil nutrients and grain yield among treatments. This analysis supported the hypothesis that soil nutrient content and availability directly affect rice grain yield while soil bacteria indirectly affect grain yield through microbial biomass production and nutrient levels. Overall, the findings of this research suggest that the integrated application of CF and manure is a better approach for improving soil health and rice yield.

## Highlights

- Manure plus mineral fertilizer improved rice yields and enhanced soil bacterial community composition in a dual cropping system.
- All of the soil bacterial communities in soils were dominated by Acidobacteria, Proteobacteria, and Chloroflexi.
- Manure inputs enhanced soil microbial-derived C and N relative to chemical fertilization.
- Soil pH and soil organic C were the keys to regulating soil microbial community structure.

## Introduction

Rice (*Oryza sativa* L.) is grown worldwide and is a staple food for more than 65% of the Chinese (Chauhan et al., 2017). An appropriate fertilization plan and sufficient soil quality are required for high crop production. To enhance crop production, farmers have increased the application of CFs. Although CFs, particularly nitrogen (N), has been one of the main factors driving rice yield enhancements over the last four decades, the low N-use efficiency caused by N overuse has led to serious environmental problems (e.g., soil acidification, degradation, biodiversity loss, water eutrophication, declining soil productivity, and increasing greenhouse gas emissions) that have attracted global attention (Duan et al., 2019; Zhang et al., 2019). A change in the source of N fertilizer is urgently needed to reduce the negative effects of CF. However, more research is needed to identify methods for increasing soil fertility, achieving sustainable and stable rice production, and reducing the use of synthetic N.

Prior studies have demonstrated that an effective way of improving soil health and crop productivity is to use a combination of manure and CFs (Iqbal et al., 2019; Oladele et al., 2019). Manure application can change a soil’s physical and biochemical properties, improve soil enzymatic activity, and decrease or eliminate harmful impacts of the CF-only overuse on soil health (Gu et al., 2015; Qaswar et al., 2020). Moreover, manure could deliver further benefits over synthetic fertilizers, such as improving soil structure, fertility, and sustaining soil health (Ali et al., 2020a; Luo et al., 2020) without sacrificing, and sometimes increasing, crop yields and grain quality (Cercioglu, 2017; Ali et al., 2020b). The increase in grain yield induced by manure fertilization is mainly due to enhancements in soil nutritional status, environmental variables, and microbial community composition (Jarvan et al., 2014; Zhang et al., 2021). However, these previous investigations were performed on a weight basis rather than the fertilization of organic fertilizer on a specific N percentage integrated with CFs in the paddy field. This has developed a gap in examining the influence of combined organic and inorganic nitrogen fertilizer on soil biochemical properties and microbial population based on particular concentration instead of weight.

Soil microorganisms play an important role in farming systems by controlling biogeochemical processes such as organic matter decomposition, nitrogen mineralization, and recycling (Xu et al., 2013), as well as promoting plant nutrient uptake and aboveground plant growth (Harris, 2009; Chaparro et al., 2012; Wagg et al., 2019). The soil bacterial community is critical for soil fertility and function due to their involvement in decomposing organic residues, releasing enzymes into the soil, recycling nutrients, and water (Ingham, 2009;Stroobants et al., 2012). Fertilizer application can alter belowground bacterial communities by changing soil’s physical and biochemical characteristics (Yu et al., 2019; Li P. et al., 2020). Therefore, belowground microorganisms are influenced by fertilizer application and can impact plant growth by altering soil nutrient turnover and status (van der Heijden et al., 2008; Wang et al., 2021). The regulation and improvement of soil microbial community diversity and activity may thus serve as a potential fertilization approach for improving soil health and crop yield while limiting the negative environmental impact of synthetic N fertilization, thus contributing to the sustainability of the farming system.

The structure and composition of soil bacterial communities require time to stabilize (Hartmann et al., 2015). Understanding how soil bacterial communities react to continuous fertilization is crucial for directing the development of an effective rice field fertilization plan. Prior studies have reported that continued fertilization of organic manure alone or combined with CF can increase soil microbial communities’ diversity and abundance and improve soil quality (Kumar et al., 2017; Cui et al., 2018; Pérez-Valera et al., 2022). Appropriate manure application can regulate the soil microbial community and improve the soil’s micro-ecological environment (Ikoyi et al., 2020; Li T. et al., 2020; Hou et al., 2022). Cui et al. (2018) found that applying organic fertilizer combined with CFs enhanced the abundance of Chloroflexi, Proteobacteria, Actinobacteria, Planctomycetes, and Firmicutes. Some research has concentrated on dryland soil agro-ecosystems (Wei et al., 2017), and only a few studies have published bacterial community characteristics in paddy soils. However, the effect of combined treatment of organic fertilizer and CFs on the bacterial community structure and its relationships with soil environmental variables in paddy fields have not been examined. Thus, characterize the variations in soil microbial biomass and bacterial community structure under different fertilizer treatments before implementing novel fertilization strategies to improve soil ecosystem health and plant productivity.

The double-rice cropping pattern is an important rice cultivation system in southern China, where low rice yields and high CFs application are common (Zhu et al., 2019). This study is based on an ongoing experiment in a rice field, with the following objectives: (i) explore the effect of organic and chemical N fertilization on rice yield, soil properties, and bacterial community structure (ii) examine the influence of integrated fertilization on the correlation among soil bacterial community and soil biochemical traits; and (iii) assess the contributions of organic–inorganic N fertilization, soil properties, and microbes to increase in rice yield. We assumed for the current study that animal manure combined with CFs could enhance soil biochemical traits and bacterial community composition, which has a positive role in higher soil quality and rice grain yield. The main objective of this study is to provide a theoretical background for scientific fertilization practices and reasonable rice cultivation while aiming to reduce chemical fertilizer (CF) use and environmental degradation.

## Materials and Methods

### Experimental Site

The field study at the rice research station of Guangxi University, Nanning, China, was started in 2019. This location has a sub-tropical monsoon climate, with approximately 1,300 mm of annual rainfall and an average annual temperature of 24.6°C. The soil is defined as Ultisols (United States Department of Agriculture soil classification) and is acidic (pH 5.96). A soil test showed that the total nitrogen (TN) was 1.66 g kg^–1^ and soil organic carbon (SOC) was 18.74 g kg^–1^; other soil properties are provided in Table 1.

**TABLE 1:** Soil and organic manure physical and chemical properties before the experimentation.

Properties	Soil	Cattle	Poultry
		manure	manure
pH (water)	5.97	7.83	7.99
Moisture content (%)	12.03	–	–
Bulk density (g cm^–3^)	1.34	0.80	0.74
SOC (g kg^–1^)	18.74	161	145
Total N (g kg^–1^)	1.66	9.57	12.98
Total P (g kg^–1^)	0.85	11.10	9.35
Total K (g kg^–1^)	–	13.83	10.06
Available N (mg kg^–1^)	158	–	–
Available P (mg kg^–1^)	27.83	–	–
Available K (mg kg^–1^)	26.73	–	–

*N, nitrogen; SOC, soil organic carbon; P, phosphorous; K, potassium.*

### Experimental Setup

The dual cropping seasons ranged from March to July (early season) and July to November (late season). The experimental study was established in a randomized complete block design with three replications. Organic manure [cattle manure (CM) and poultry manure (PM)] and CF urea were used, and the combinations of treatment were: no N fertilizer control (Neg-Con); 100% CF in the form of urea (Pos-Con); 60% CM + 40% CF (High-CM); 30% CM + 70% CF (Low-CM); 60% PM + 40% CF (High-PM), and 30% PM + 70% CF (Low-PM). We grew the Zhenguiai, a broadly cultivated cultivar in Guangxi Province. This cultivar has a short growth duration (approximately 110–120 days) with a morphological structure and high grain filling rate (Li et al., 2006). Rice seeds were grown, and 25-days old seedlings of even size were transferred into the fields. Except for Neg-Con, which received no N fertilizer amendments, the recommended dose of N, P, and K at a rate of 150:75:150 (kg ha^–1^) was applied to each plot (Sapkota et al., 2020). Table 2 shows the nutrient content of manure and the amount of each treatment. The potassium (KCl) and N fertilizers were delivered in three stages: 50% prior to transplantation, 30% during tillering, and the remaining 20% during the heading period. All phosphorous fertilizer was supplied as a base dose before transplanting. Furthermore, uniform flooding continued from seedling transplantation to rice physiological maturity. Normal agricultural practices, such as irrigation and insecticide application, were performed for all regimes.

**TABLE 2:** Content and amount of NPK provided of each plot and fertilization time.

Treatment	N (kg/ha)	Urea (kg/ha)	CM and PM (kg/ha)	Basal fertilization (kg/ha)	Tillering (kg/ha)	Panicle initiation (kg/ha)
Neg-Con: N = 0% (T_1_)	0	0	0	P_2_O_2_: 397, KCl: 128	KCl: 128	Urea: 0
Pos-Con: 100% CF (T_2_)	150	322	0	Urea: 192, P_2_O_2_: 397, KCl: 128	Urea: 65, KCl: 128	Urea: 65
High CM: 60% CM + 40% CF (T_3_)	150	130	9,188	Urea: 0, CM: 9188, P_2_O_2_: 397, KCl: 128	Urea: 65, KCl: 128	Urea: 65
Low CM: 30% CM + 70% CF (T_4_)	150	225	4,572	Urea: 94, CM: 4572, P_2_O_2_: 397, KCl: 128	Urea: 65, KCl: 128	Urea: 65
High PM: 60% PM + 40% CF (T_5_)	150	128	6,623	Urea: 0, PM: 6623, P_2_O_2_: 397, KCl: 128	Urea: 65, KCl: 128	Urea: 65
Low PM: 30% PM + 70% CF (T_6_)	150	225	3,290	Urea: 94, PM: 3290, P_2_O_2_: 397, KCl: 128	Urea: 65, KCl: 128	Urea: 65

*CF, chemical fertilizer; CM, cattle manure; PM, poultry manure; N, nitrogen; KCl, potassium chloride.*

### Soil Sampling and Analysis

#### Soil Properties

Soil samples were collected using a core sampler at depth (0–20 cm) from each plot post rice harvest in 2019–2020. Samples were obtained from various places in each plot and then combined to produce a mixture, separated into two parts: one for nutrient measurements and the other for molecular examination. Soil organic C (SOC) was measured using the K_2_Cr_2_O_7_-H_2_SO_4_ oxidation method as described by Wang et al. (2003). In addition, for total N, 200 mg of the soil samples were treated according to Ohyama et al. (1991). Finally, TN was determined by the micro-Kjeldahl method as recommended by Jackson (1956). For soil available N, P, and K and pH were assessed by the procedures of Lu (2000). The fumigation extraction technique was used to examine microbial biomass carbon (MBC) as described by Brookes et al. (1985) and microbial biomass nitrogen (MBN) by the technique of Vance et al. (1987).

#### Extraction of DNA and PCR Amplification

DNA was extracted from wet soil samples (0.25 g) following the guidelines of the E.Z.N.A. Soil DNA Kit of the Omega, United States. Extracted DNA (50 ng/reaction) was used as a template in standard 16S ribosomal RNA (rRNA) gene amplification targeting the V3–V4 variable regions of microbial 16S rRNA genes, as described previously (Sundberg et al., 2013). The primers used included 341F (CCTACGGGNGGCWGCAG) and 805R (GACTACHVGGGTATCTAATCC) (Klindworth et al., 2013). Primers contained Illumina sequencing adapters and sample-specific barcodes. PCR reactions were performed using mastermix, with the following cycling conditions: 94°C for 3 min, followed by 5 cycles at 94°C for 30 s, 45°C for 20 s, 65°C for 30 s, and then 20 cycles at 94°C for 20 s, 55°C for 20 s, 72°C for 30 s with a final extension of 72°C for 5 min. Library preparation and sequencing were performed by Illumina MiSeq system (Illumina MiSeq, United States) (Claesson et al., 2010).

#### Illumina MiSeq Sequencing

After PCR, amplicons of the correct size were extracted from 2% agarose gels and purified by an AxyPrep DNA Gel Extraction Kit (Axygen Biosciences, CA, United States). The amplicons were quantified *via* Qubit 3.0 fluorometer (Thermo Fisher Scientific, Waltham, MA, United States). Amplicons were pooled in equimolar levels and sequenced on an Illumina MiSeq sequencer (Illumina, San Diego, CA, United States) following standard sequencing protocols.

#### Processing of Sequencing Data

Raw paired-end sequence data were merged using the PEAR software package (Zhang et al., 2014). Merged sequence data were processed through the software package QIIME (Caporaso et al., 2010). UCLUST (Edgar, 2010) was used to identify operational taxonomic units (OTUs) based on 97% similarity thresholds (Robert et al., 2010). Chimeric sequences were identified and removed using the USEARCH tool (Edgar, 2010). Taxonomic classification was carried out on representative sequences from each OTU using the SILVA 119 reference database (Quast et al., 2012) using the RPD classifier (Cole et al., 2009) or UCLUST with a 90% confidence threshold level.

### Rice Grain Yield

At maturity, the rice was collected from the entire plot and the grain yields. The dry weight of the grain was calculated using an adjusted moisture content of 14% in rice grains.

### Statistical Analysis

The changes in soil biochemical characteristics and rice grain yields as affected by fertilization treatment were examined using the ANOVA method in Statistics 8.1 (Analytical Software Tallahassee, FL, United States). First, the data were subjected to standard tests to ensure they met the assumptions of normality. Furthermore, percentages data were arcsine transformed before analysis to normalize the variables. Tukey’s *post hoc* test was performed to compare means for the variables where the effects of treatments were significant. A Venn diagram was used to illustrate the number of related and distinctive OTUs in the samples and determine the similarity and coincide in the number of OTUs between the samples. Microbial alpha diversity indices, including Simpson’s and Shannon indices, were calculated using MOTHUR (Schloss et al., 2009). Species richness rarefaction curves were plotted against the series of data using the MicrobiomeAnalyst (Dhariwal et al., 2017). RDA was used to analyze the relationship strength between soil traits and soil bacterial diversity using the software package CANOCO5 (Microcomputer Power, Ithaca, NY, United States).

## Results

### Impact of Fertilization on Soil Biochemical Attributes

Co-fertilization of fields with manure and chemical nutrients significantly increased the SOC, TN, pH, MBC, and MBN compared to CF application: Pos-Con and Neg-Con (Table 3). The effect was greatest in all observed data when manure input was high; no substantial (*P* < 0.05) variations among CM and PM were observed. Across the seasons, the regimes revealed the same trend. Compared to Pos-Con, High CM and PM treatments increased SOC 26.3 and 31.2%, TN 25.3 and 25.6%, respectively. In addition, over the years, High-CM enhanced treatment increased soil MBN and MBC by 55 and 61%, respectively, relative to Pos-Con. Nevertheless, High CM was statistically (*P* < 0.05) comparable to High PM. Likewise, low manured input treatments also increased the measured soil biochemical traits as compared to control.

**TABLE 3:** Changes in soil biochemical traits under the combined manure and mineral N fertilization.

		pH (water)	SOC (g kg^–1^)	TN (g kg^–1^)	MBC (mg kg^–1^)	MBN (mg kg^–1^)
Year	Treatment					
2019	Neg-Con	5.93c	14.74b	1.40c	170d	23.34d
	Pos-Con	5.91c	15.08b	1.41c	220c	31.44c
	High-CM	6.25a	19.24a	1.78a	340a	44.67a
	Low-CM	6.12b	16.81b	1.67b	310b	38.43b
	High-PM	6.23a	18.92a	1.77a	341a	43.89a
	Low-PM	6.13b	16.88b	1.64b	315b	39.44b
2020	Neg-Con	5.95d	14.88c	1.40d	175d	24.52e
	Pos-Con	5.94d	15.52c	1.44c	235c	34.45d
	High-CM	6.31a	24.74a	1.99a	370a	51.42ab
	Low-CM	6.18c	22.25b	1.81b	355b	43.36c
	High-PM	6.28ab	25.05a	1.98a	374a	54.58a
	Low-PM	6.16c	21.77b	1.80b	311b	41.35c

*SOC, soil organic carbon; TN, total nitrogen; MBC, microbial biomass carbon; MBN, microbial biomass nitrogen; Neg-Con, control; Pos-Con, 100% chemical fertilizer (CF); High-CM, 60% cattle manure (CM) + 40% (CF); Low-CM, 30% CF + 70% CF; High-PM, 60% poultry manure (PM) + 40% CF; Low-PM, 30% PM + 70% CF.*

*The mean comparison was made using Tukey tests for treatment mean in both years and the lettering was done based on the Tukey HSD test at 5% using a simple effect.*

*Values followed by the same letters within the column are statistically the same at P ≤ 0.05.*

In addition, significant increases were measured for SOC, TN content, MBC, and MBN in the subsequent year; the average enhancement in SOC, TN, MBC, and MBN during 2020 was 26.4, 9.50, 8.9, and 22%, respectively, relative to 2019.

### Impact of Combined Fertilization on Bacterial Diversity and Abundance Index

**FIGURE 1:**
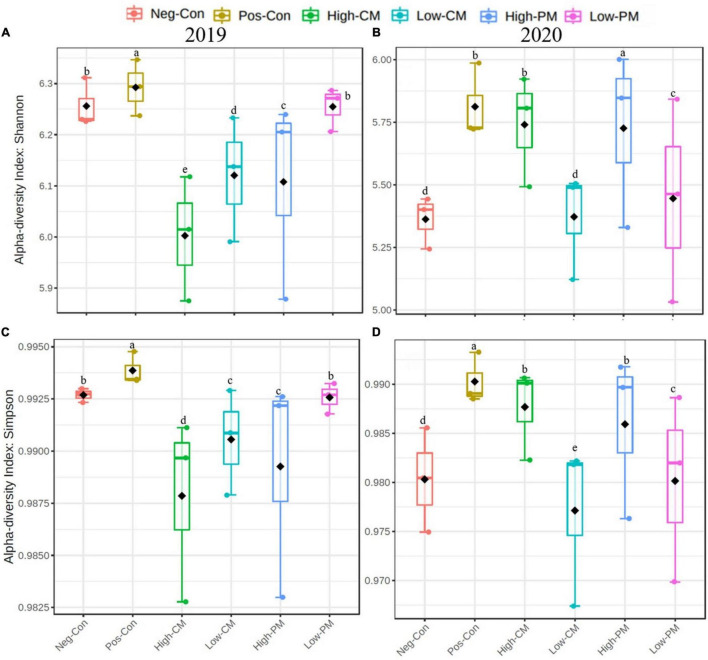
Comparison of the soil bacterial communities based on PCR-amplified 16S rRNA gene analysis. Box plots for α-diversity estimated Shannon **(A,B)** and Simpson **(C,D)** indices of the bacterial community under six treatments. The ends of the whiskers represent minimum and maximum, the bottom and top of the box are the first and third quartiles, and the black dot inside the box is the median. Bars show the standard error of the mean, and bars with different letters are significantly different at *P* < 0.05. Please see Table 3 for the treatment combination.

In this study, the co-applied manure and synthetic N significantly influenced the soil bacterial community composition and diversity (Figures 1A–H). The box plots based on the Simpson and Shannon index exhibited significant variations in bacterial α-diversity detected in the regimes. Co-fertilization of manure and CF resulted in the highest bacterial diversity and abundance relative to Pos-Con. In addition, the bacterial diversity and abundance were significantly higher in the High manured plots.

### Impact of Combined Fertilization on Soil Bacterial Community Diversity and Composition

**FIGURE 2:**
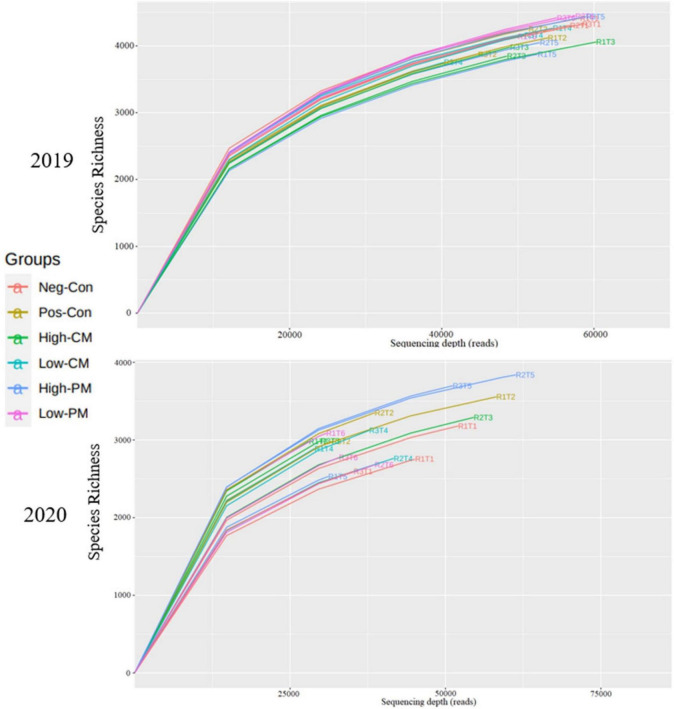
Rarefaction curves of 16S rRNA sequencing depth and number of species numbers in soil depth (0–20 cm). Neg-Con (R1T1, R2T1, and R3T1), Pos-Con (R1T2, R2T2, and R3T2), High-CM (R1T3, R2T3, and R3T3), Low-CM (R1T4, R2T4, and R3T4), High-PM (R1T5, R2T5, and R3T5), and Low-PM (R1T6, R2T6, and R3T6). See Table 3 for the treatments combination.

**FIGURE 3:**
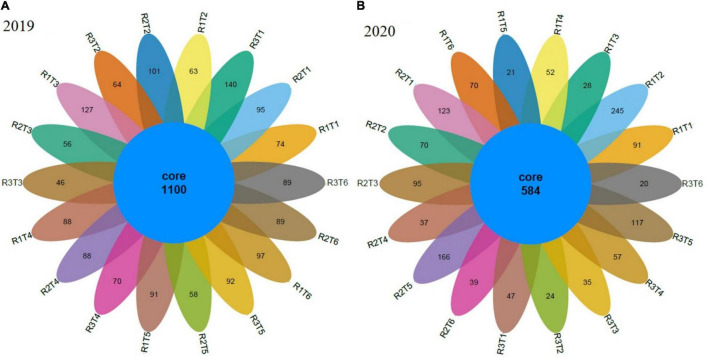
Venn diagram showing the bacterial unique and operational units (OTUs) during 2019 **(A)** and 2020 **(B)** under organic and inorganic fertilization. Neg-Con (R1T1, R2T1, and R3T1), Pos-Con (R1T2, R2T2, and R3T2), High-CM (R1T3, R2T3, and R3T3), Low-CM (R1T4, R2T4, and R3T4), High-PM (R1T5, R2T5, and R3T5), and Low-PM (R1T6, R2T6, and R3T6). See Table 3 for the treatments combination.

To determine rarefaction curves, richness, and diversity of bacteria, OTUs were identified at 97% of genetic similarity. The rarefaction curves exhibited that the sequencing effort was enough to describe most of the diversity in soil samples (Figure 2). The Venn diagram shows that in 2019, the number of unique OTUs in Neg-Con, Pos-Con, High-CM, Low-CM, High-PM, and Low-PM treatments was 309, 228, 229, 246, 241, and 275, correspondingly, and the number of shared OTUs was 1,100 (Figure 3). In 2020, the number of unique OTUs was 261, 339, 158, 146, 304, and 129, respectively, and the number of mutual OTUs was 584.

**FIGURE 4:**
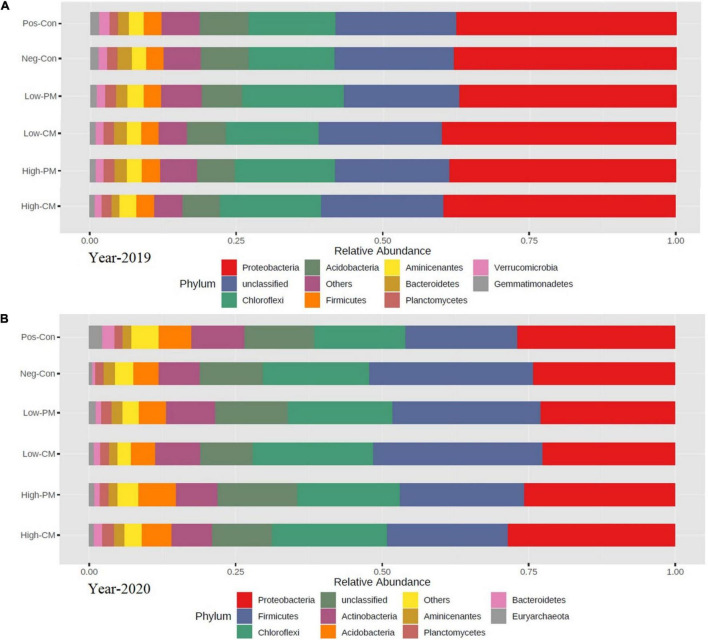
Based on the 16S rRNA gene the relative abundance of soil bacterial community composition at phylum level during year 2019 **(A)** and year 2020 **(B)** in six treatments. Each strip represents the mean of three replicates. See Table 3 for treatments combination.

**FIGURE 5:**
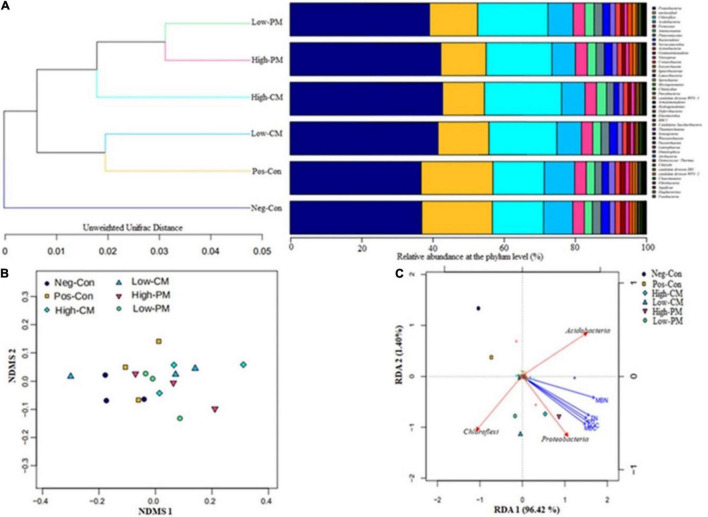
The similarity between soil bacterial communities of different fertilizers treatments is confirmed by cluster analysis **(A)**, NDMS analysis based on unweighted Unifrac distance at OTU levels **(B)**, and redundancy ordination analysis (RDA) **(C)** showing the strength of association between the different treatments, environmental factors (represented by yellow arrows), and dominant bacteria at the phylum level (represented by red arrows). For fertilization treatment combination details, see Table 3.

The analysis for bacterial community composition under combined manure and mineral fertilization indicated the presence of a total of 41 phyla, and the average relative abundance of 11 phyla exceeded 1% (Figures 4, 5). Compared to Pos-Con, the integrated application of manure with mineral fertilizer significantly increased the soil bacterial community composition. The top five dominant phyla in all treatments were Chloroflexi, Proteobacteria, Firmicutes, Acidobacteria, and Planctomycetes, which reported more than 70% of the relative abundance of the bacterial communities. Chloroflexi and Proteobacteria were the most abundant among all these dominant phyla in the High manured treatments compared to the Neg-Con and Pos-Con. Likewise, low CM and PM treatments also increased the abundance of Proteobacteria and Chloroflexi compared with control. Furthermore, Actinobacteria, Acidobacteria, and Firmicutes were the second most abundant phyla in the experiment across all treatments.

The cluster analysis (Figure 5A) demonstrates that bacterial communities in the six fertilization regimes are divided into main groups. One is made up of Neg-Con, the other is composed of High-CM, High-PM, Low-CM, Low-PM, and Pos-Con, indicating that bacterial communities in different fertilization regimes share a high similarity and the cluster. Moreover, the similarity among bacterial communities of all fertilization regimes is further proved by the NDMS analysis as shown in Figure 5B. In the two-dimensional NDMS plot, a small unweighted UniFrac distance indicates a similar bacterial community. As projected, soil samples in Neg-Con and Pos-Con are closely occurred and clustered and separated from other manured amendments along NMDS axes.

### Impact of Combined Fertilization on Rice Yield

**FIGURE 6:**
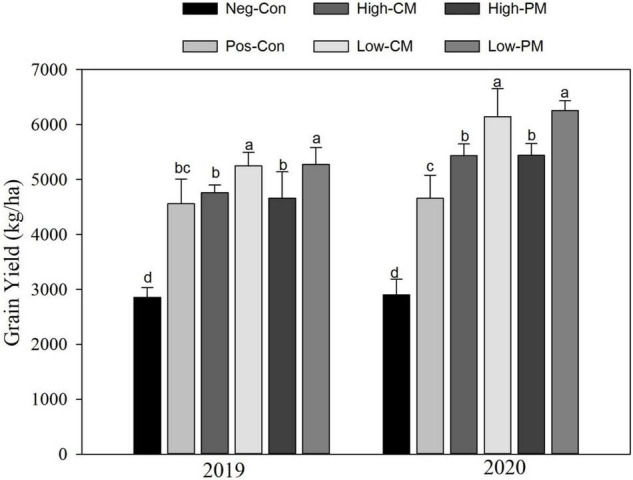
Variation in rice yield as affected by organic and inorganic N application. The mean comparison was made using the Tukey’s *post hoc* test for treatments at 5%. Different letters on bars are not significantly different at *P* < 0.05. See Table 3 for treatments combination.

The joined manure and synthetic N fertilization considerably affected the rice yield in both years (Figure 6). Combined fertilization increased rice yield considerably across the years compared with control, and over both years, the regimes exhibited a similar trend. The Low PM treatment increased the grain yield by 17 and 32% in 2019 and 2020, correspondingly compared to the control. Nevertheless, there were no substantial differences (*P* < 0.05) in grain yield between the Low manured regimes. Likewise, High manured treatments also significantly enhanced rice yield relative to the Pos-Con. Significant enhancements in yield were noticed over the years, and the average rice yield was improved by 15% in 2020 relative to 2019.

### Relationship Between Fertilization Treatments, Soil Traits, and Microorganisms

Redundancy analysis was performed to determine the impact of different fertilization treatments on bacterial community composition and environmental factors and reveal the strength of the relationship between soil traits and soil bacterial community structure (Figure 5C). The RDA shows that the six treatments occurred in distant quadrants show that the treatments had a significant effect on the structure of soil microorganisms and soil properties. The RDA revealed that manure-treated treatments significantly affected soil bacterial community composition compared to non-manure treatments. Proteobacteria, Acidobacteria, and Chloroflexi were strongly associated with soil properties. The RDA outcomes demonstrated that combined organic and inorganic fertilizer application to the rice field has the most substantial influence on soil microbial community composition and environmental properties.

## Discussion

This study investigated the effect of continued manure substitution on rice field soil biochemical traits and bacterial community structure and composition. The microbial population is the most important indicator of soil biology, as it is responsible for improving soil fertility, health, and crop yield (Luo et al., 2020; Liu et al., 2021).

### Soil Properties

In this experiment, animal manure combined with CFs significantly increased soil qualitative traits (SOC, TN, MBC, MBN, and pH) compared with CF-only fertilization (Table 3). We detected that the biodegradation of animal manure gradually released plant nutrients to the soil and exhibited that increasing the rate of manure improves soil qualitative traits. Application of CFs reduced soil pH level, while joint manure amendments increased soil pH level significantly. Alike results were stated by Bhattacharyya et al. (2015), who concluded that continuous use of synthetic fertilizer causes soil acidification. Moreover, the decline in pH level is because the overuse of synthetic N forms H^+^ through nitrification in soil (Yang et al., 2018). Another possible reason for the lower pH resulting from the mineral N fertilization was the acidic nature of mineral N fertilizers, which could donate to the soil’s lower pH (Adekiya et al., 2020). Organic fertilizer application alters soil acidification because it frequently consists of enough basic cations and carbonate ions to neutralize soil acidity, explaining the increase in soil pH under manure amendment plots (Duruigbo et al., 2007).

The application of animal manure and CF meaningfully enhanced the SOC and TN, P and K status in the paddy soil (Table 3), which is in line with Wei et al. (2017). The main macronutrients (N, P, and K) are important for plant growth and production. Soil N, P, and K scarcity have a negative impact on soil fertility and crop yield (Kaur and Reddy, 2014; Verma et al., 2017). The substantial improvement of soil organic C (SOC) could be associated to the substantial effects of manure fertilization because the soil C variation rate is affected by direct carbon inputs from organic fertilizer and indirect carbon inputs from increased plant biomass return (Bitew and Alemayehu, 2017; Wang et al., 2021), such as crop and root residues. This is primarily due to the addition of manure (i.e., CM or poultry) having a significant effect on the soil TN, TP, and TK. This increment in nutrient content might also be related to incorporating manure remains, which, after decomposition, directly added nutrients to the soil (Zhang et al., 2019; Hou et al., 2022). Moreover, besides improving the physical and biochemical traits of soil, animal manure fertilization, steady releases of plant nutrients and prevent nutrient losses from the synthetic fertilizers by binding to plant nutrients and releasing them (Abedi et al., 2010; Ullah et al., 2020). Thus, the integrated application of manure and CFs improves CF use efficiency and, hence, decreases CF’s rate (Tilahun-Tadesse et al., 2013; Adekiya et al., 2019).

In the present research, organic fertilizer in conjunction with synthetic fertilizer, increased microbial biomass C and N (Table 3). Rises in MBC and MBN may have happened because manure enhanced the biogeochemical properties of the soil in the present study, leading to improved absorption of inorganic N by the plant (Ahmad et al., 2008). Another reason is that organic amendments may have boosted soil nutrient richness and crop biomass accumulation, resulting in enhanced crop residues (Akhtar et al., 2019). Such residues are useful for spreading soil bacteria and may promote the alteration of N and C (Lima et al., 2009).

In this experiment, the residual effects of manure have increased soil biochemical traits. Soil organic C, examined as a vital factor in evaluating soil health and quality, enhanced 26.4% in the residual effect of manure fertilization. A rise in SOC due to organic manure fertilization has a significant impact on soil productivity because SOC is the ultimate source of soil nutrients and soil microorganisms activity in the soil. Moreover, SOC also has a key role in enhancing soil quality, infiltration rate, water holding ability, porosity and aeration (Sarwar et al., 2008; Ali et al., 2020a). Moreover, manure contains macro-and micronutrients, such as N, K, P, Ca, and Mg (Abedi et al., 2010; Hafidi et al., 2012). In this study, a significant increase was noted in TN and MBN in the following year; the average enhancement in TN and MBN in 2020 was 9.5 and 22.2% compared with 2019. The observed residual effect of organic fertilizers or enhancement in nutrient contents of manured plots in our study might be due to the reason that soil nutrients contained in organic fertilizers are revered in soil for a longer period and released steadily, ensuring a long term residual influence, and to solubilization of plant nutrients from soil minerals due to the influence of organic manure organic acids (Sharma et al., 2013).

### Bacterial Community Diversity and Composition

Microbes are a key index of soil quality (van Bruggen et al., 2015). The richness and biodiversity of the microbial population are crucial to soil integrity, performance, and soil sustainability; however, they are frequently reduced by conventional agricultural techniques (Zhao et al., 2014). In the current study, the fertilization treatments extensively affected the bacterial community structure and composition, and the soil bacterial communities were influenced by the application of organic amendments (Figures 1, 4). Manure and synthetic fertilizer co-application significantly increased the number of sequences and the species richness (i.e., ACE, Shannon, and Chao 1 index values) of the bacteria related to sole inorganic fertilizer (Table 4 and Figure 1). According to Dong et al. (2014), manure applications not only include a wider range of substrates for bacterial activity than synthetic fertilizers, and they also directly introduce microorganisms found in manure into the soil. Furthermore, Bacteria are broadly considered as critical mediators of the quick pathways of nutrient cycling in soil, and their growth rate is nearly ten times higher than that of fungi (Rousk and Bååth, 2007; Schmidt et al., 2014); thereby, bacterial communities can grow rapidly and diversify under manure addition.

**TABLE 4:** Pearson correlation coefficients between soil properties, soil bacterial community, and rice grain yield.

	SOC	TN	AN	MBC	MBN	OTU
TN	0.99**					
AN	0.93**	0.92**				
MBC	0.99**	0.97**	0.92**			
MBN	0.99**	0.98**	0.92**	0.98**		
OUT	0.56*	0.27	0.12	0.62*	0.57*	
GY	0.79**	0.71*	0.71*	0.84**	0.79**	0.21

SOC, soil organic carbon; TN, total nitrogen; AN, available nitrogen; MBC, microbial biomass carbon; MBN, microbial biomass nitrogen; OUTs, operational taxonomic units; GY, grain yield. *P < 0.05, **P < 0.01.

In the current study, the bacterial community structure in synthetic fertilizers treatment and non-synthetic fertilizer treatment was similar; however, it changed following manure amendments treatment (Figure 4). This is probably because of short-term input of CFs did not considerably affect the soil pH. According to Ganzert et al. (2014), pH is the greatest important factor shaping the soil bacterial community. Furthermore, Geisseler and Scow (2014) stated that the response of CFs on soil microorganisms is carbon and pH-dependent. Organic amendments could enhance soil interspace and resource supply for bacterial growth (Li P. et al., 2020), thereby prompting microbe societies. Moreover, cluster analysis showed a substantial difference in bacterial community composition between manure treated and non-manure treatment (Figure 5A). This is primarily due to livestock manure containing more readily available microbial organic resources that can provide additional metabolic nutrients to soil microbes (Bei et al., 2018).

The integrated use of animal manure and CF increased the relative abundance of Proteobacteria, Chloroflexi, Firmicutes, Acidobacteria, and Planctomycetes, which described for more than 70% of the relative abundance of the soil bacterial communities (Figure 4). The relative abundance of the community’s dominant bacteria, Proteobacteria and a type of nutrient-rich bacteria are associated with soil properties (Merila et al., 2010; Luo et al., 2020). In our research, all soil chemical traits improved with manure fertilization (Table 3), promoting the growth of bacteria. Thus, the relative abundance of Acidobacteria, Chloroflexi, Proteobacteria, Firmicutes, and Planctomycetes, the dominant bacteria in the community, enhanced under the integration of organic and synthetic fertilizer treatments.

### Grain Yield

**FIGURE 7:**
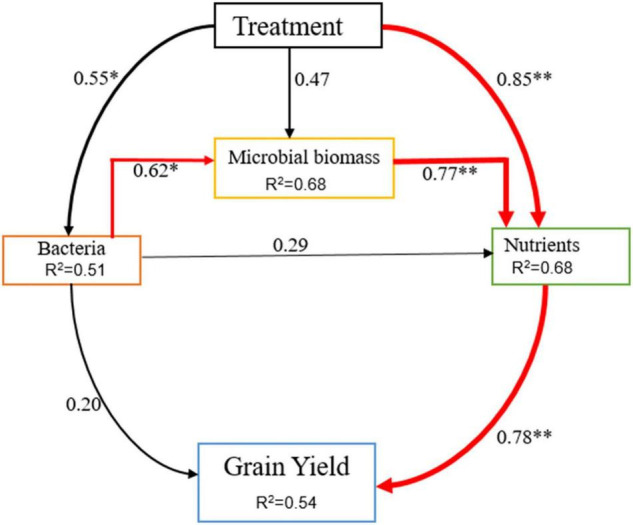
The value above the structural equation model line shows the path coefficient. The red lines represent the positive path coefficient and the black lines represent the non-significant path coefficient. The width of the arrow indicates the significance of the standard path coefficient (^**^P < 0.01 and *P < 0.05).

Application of organic and inorganic N significantly improved rice yield related to CF-only application in the current study. Manure application improves the physical and biogeochemical properties of the soil, resulting in increased plant growth and crop yield (Abedi et al., 2010; Hafidi et al., 2012). In this study, higher soil nutrients were observed under manured plots (Table 3), which, in turn, improved rice growth and biomass accumulation by providing adequate nutrients during the growth period. Moreover, The residual effect of 1 year (two consecutive seasons) of manure and CFs application also provided yield benefits. Significant increases in rice grain yield were observed between years, and the average rice yield was enhanced by 14% in the year 2020 relative to the year 2019. Person correlation analysis exhibited that grain yield was strongly correlated with SOC, TN, TP, and AK of soil (Table 4). This correlation shows that deficiency of anyone the three main nutrients (NPK) can decrease grain yield in flooded soil. The structural equation model result showed that differences in soil nutrients explained the greatest proportion of the variation in rice crop yield (Figure 7). These findings collectively show the importance of balanced and enough fertilization of organic and chemical N fertilizers for soil quality and sustainable grain yield of rice. According to Akhtar et al. (2019), changes in crop yield are closely related to soil biogeochemical characteristics and microbial biomass yield.

### Relationships Between Soil Bacterial Communities and Biochemical Traits

Manure fertilization can cause physicochemical variations in soil, resulting in changes in the bacterial community composition (Li et al., 2021). This study discovered that organic amendment treatments dramatically altered soil qualitative traits (Table 3). Moreover, Wu et al. (2020) reported that the structure and composition of the bacterial community were positively associated with soil C and N content. Figure 5C shows the relationship between the soil traits and the bacterial communities for the different fertilizer regimes. In this study, RDA showed that the organic amendments had significant effects on bacterial community and soil qualitative traits. The dominant bacteria at the phyla, notably Proteobacteria, Chloroflexi, and Acidobacteria, had a positive correlation with soil characteristics, but Proteobacteria had a strong correlation with SOC, TN, and pH. Bacterial growth is highly related to the type of fertilizer used, and controlling the type and ratio of animal fertilizer is an effective strategy for increasing bacterial growth. According to what has been discussed thus far, applying organic fertilizer combined with reduced inorganic fertilizers could provide a faster growth environment for soil microbes, improving the bacterial population structure and soil fertility.

## Conclusion

Poor soil quality and fertility due to excessive use of CFs is a major threat to agricultural sustainability and food security. Organic fertilizer application as a substitute for CF is an adequate fertilization approach for preserving soil health and biodiversity. In the current study, CF combined with cattle or PM improved soil fertility, bacterial community diversity and composition, and rice grain yield compared to the sole use of CF. A comparison of the structure of the bacterial community and its relationship to soil properties in manured regimes, we noted that manure application enhanced soil nutrients and decreased soil acidification, which contributed directly to higher rice yield. Furthermore, the substitution of animal manure regulated the relationship between soil bacteria and soil biochemical traits, influencing the soil microbial population. Our findings demonstrated that soil biochemical indicators play an important role in forming the structure and stability of soil bacterial communities. Our results indicated that combining manure and CFs applications based on nutrient levels and proper fertilizers composition is the most effective approach for improving bacterial community structure and soil fertility, ensuring yield sustainability.

## Data Availability Statement

The datasets presented in this study can be found in the NCBI under accession number PRJNA822677.

## Author Contributions

AI and LJ: conceptualization and writing—original draft. AI, IA, and PY: methodology and formal analysis. AI, IA, AK, ZH, and LH: investigation. LJ and SW: resources and supervision. AI, AK, ZH, LH, and PY: data curation. PY, AK, and LJ: writing—review and editing. All authors contributed to the article and approved the submitted version.

## Conflict of Interest

The authors declare that the research was conducted in the absence of any commercial or financial relationships that could be construed as a potential conflict of interest.

## Publisher’s Note

All claims expressed in this article are solely those of the authors and do not necessarily represent those of their affiliated organizations, or those of the publisher, the editors and the reviewers. Any product that may be evaluated in this article, or claim that may be made by its manufacturer, is not guaranteed or endorsed by the publisher.

## Abbreviations

OF: organic fertilizer
PM: poultry manure
CF: chemical fertilizer
CM: cattle manure
TN: total nitrogen
SOC: soil organic carbon
AN: available nitrogen
MBC: microbial biomass carbon
OTUs: operational taxonomic units.

## References

[B1] Abedi T. Alemzadeh A. KazemeIni S. A. (2010). Effect of organic and inorganic fertilizers on grain yield and protein banding pattern of wheat. *Aust. J. Crop Sci.* 4 384–389.

[B2] Adekiya A. O. Agbede T. M. Aboyeji C. M. Dunsin O. Simeon V. T. (2019). Effects of biochar and poultry manure on soil characteristics and the yield of radish. *Sci. Hortic*. 243 457–463. 10.1016/j.scienta.2018.08.048

[B3] Adekiya A. O. Agbede T. M. Ejue W. S. Aboyeji C. M. Dunsin O. Aremu C. O. (2020). Biochar, poultry manure and NPK fertilizer: sole and combine application effects on soil properties and ginger (Zingiber officinale Roscoe) performance in a tropical Alfisol. *Open Agric.* 5 30–39. 10.1515/opag-2020-0004

[B4] Ahmad R. Arshad M. Khalid A. Zahir Z. A. (2008). Effectiveness of organic-/bio-fertilizer supplemented with chemical fertilizers for improving soil water retention, aggregate stability, growth and nutrient uptake of maize (*Zea mays* L.). *J. Sustain. Agric*. 31 57–77. 10.1300/J064v31n04_05

[B5] Akhtar K. Wang W. Khan A. Ren G. Zaheer S. Sial T. (2019). Straw mulching with fertilizer nitrogen: an approach for improving crop yield, soil nutrients and enzyme activities. *Soil Use Manag.* 35 526–535. 10.1111/sum.12478

[B6] Ali I. He L. Ullah S. Quan Z. Wei S. Iqbal A. (2020a). Biochar addition coupled with nitrogen fertilization impacts on soil quality, crop productivity, and nitrogen uptake under double-cropping system. *Food Energy Sec.* 9:e208. 10.1002/fes3.208

[B7] Ali I. Ullah S. He L. Zhao Q. Iqbal A. Wei S. (2020b). Combined application of biochar and nitrogen fertilizer improves rice yield, microbial activity and N-metabolism in a pot experiment. *PeerJ* 8:e10311. 10.7717/peerj.10311 33240639 PMC7668215

[B8] Bei S. Zhang Y. Li T. Christie P. Li X. Zhang J. (2018). Response of the soil microbial community to different fertilizer inputs in a wheat-maize rotation on a calcareous soil. *Agri. Ecosyst. Environ.* 260 58–69. 10.1016/j.agee.2018.03.014

[B9] Bhattacharyya R. Ghosh B. N. Mishra P. K. Mandal B. Rao C. S. Sarkar D. (2015). Soil degradation in India: challenges and potential solutions. *Sustainability* 7 3528–3570. 10.3390/su7043528

[B10] Bitew Y. Alemayehu M. (2017). Impact of crop production inputs on soil health: a review. *Asian J. Plant Sci.* 16 109–131. 10.3923/ajps.2017.109.131

[B11] Brookes P. C. Landman A. Pruden G. Jenkinson D. (1985). Chloroform fumigation and the release of soil nitrogen: a rapid direct extraction method to measure microbial biomass nitrogen in soil. *Soil Biol. Biochem*. 17 837–842. 10.1016/0038-0717(85)90144-0

[B12] Caporaso J. G. Kuczynski J. Stombaugh J. Bittinger K. Bushman F. D. Costello E. K. (2010). QIIME allows analysis of high-throughput community sequencing data. *Nat. Methods* 7 335–336. 10.1038/nmeth.f.303 20383131 PMC3156573

[B13] Cercioglu M. (2017). The role of organic soil amendments on soil physical properties and yield of maize (*Zea mays* L.). *Commun. Soil Sci. Plant Anal.* 48 683–691. 10.1080/00103624.2017.1298787

[B14] Chaparro J. M. Sheflin A. M. Manter D. K. Vivanco J. M. (2012). Manipulating the soil microbiome to increase soil health and plant fertility. *Biol. Fertil. Soils* 48 489–499. 10.1007/s00374-012-0691-4

[B15] Chauhan B. S. Jabran K. Mahajan G. (eds) (2017). *Rice Production Worldwide*, Vol. 247. Cham: Springer International Publishing. 10.1007/978-3-319-47516-5

[B16] Claesson M. J. Wang Q. O’Sullivan O. Greene-Diniz R. Cole J. R. Ross R. P. (2010). Comparison of two next-generation sequencing technologies for resolving highly complex microbiota composition using tandem variable 16S rRNA gene regions. *Nucleic Acids Res.* 38:e200. 10.1093/nar/gkq873 PMC3001100 20880993

[B17] Cole J. R. Wang Q. Cardenas E. Fish J. Chai B. Farris R. J. (2009). The ribosomal database project: improved alignments and new tools for rRNA analysis. *Nucleic Acids Res.* 37(Suppl. 1) D141–D145. 10.1093/nar/gkn879 19004872 PMC2686447

[B18] Cui X. W. Zhang Y. Z. Gao J. S. Peng F. Y. Gao P. (2018). Long-term combined application of manure and chemical fertilizer sustained higher nutrient status and rhizospheric bacterial diversity in reddish paddy soil of central South China. *Sci. Rep*. 8:16554. 10.1038/s41598-018-34685-0 30410029 PMC6224536

[B19] Dhariwal A. Chong J. Habib S. King I. L. Agellon L. B. Xia J. (2017). Microbiome analyst: a web-based tool for comprehensive statistical, visual and meta-analysis of microbiome data. *Nucleic Acids Res.* 45 W180–W188. 10.1093/nar/gkx295 28449106 PMC5570177

[B20] Dong W. Y. Zhang X. Y. Dai X. Q. (2014). Changes in soil microbial community composition in response to fertilization of paddy soils in subtropical China. *Appl. Soil Ecol*. 84 140–147. 10.1016/j.apsoil.2014.06.007

[B21] Duan P. Fan C. Zhang Q. Xiong Z. (2019). Overdose fertilization induced ammoniaoxidizing archaea producing nitrous oxide in intensive vegetable fields. *Sci. Total Environ*. 650 1787–1794. 10.1016/j.scitotenv.2018.09.341 30278423

[B22] Duruigbo C. I. Obiefuna J. C. Onweremadu E. U. (2007). Effect of poultry manure rates on the soil acidity in an Ultisol. *Int. J. Soil Sci.* 2 154–158. 10.3923/ijss.2007.154.158

[B23] Edgar R. C. (2010). Search and clustering orders of magnitude faster than BLAST. *Bioinformatics* 26 2460–2461. 10.1093/bioinformatics/btq461 20709691

[B24] Ganzert L. Bajerski F. Wagner D. (2014). Bacterial community composition and diversity of five different permafrost-affected soils of Northeast Greenland. *FEMS Microbiol. Ecol.* 89 426–441. 10.1111/1574-6941.12352 24819653

[B25] Geisseler D. Scow K. M. (2014). Long-term effects of mineral fertilizers on soil microorganisms–a review. *Soil Biol. Biochem.* 75 54–63. 10.1016/j.soilbio.2014.03.023

[B26] Gu B. Ju X. Chang J. Ge Y. Vitousek P. M. (2015). Integrated reactive nitrogen budgets and future trends in China. *Proc. Natl. Acad. Sci. U.S.A.* 112:8792. 10.1073/pnas.1510211112 26124118 PMC4507225

[B27] Hafidi M. Amir S. Meddich A. Jouraiphy A. Winterton P. El Gharous M. (2012). Impact of applying composted biosolids on wheat growth and yield parameters on a calcimagnesic soil in a semi-arid region. *Afr. J. Biotech.* 11 9805–9815. 10.5897/AJB10.1010

[B28] Harris J. (2009). Soil microbial communities and restoration ecology: facilitators or followers? *Science* 325 573–574. 10.1126/science.1172975 19644111

[B29] Hartmann M. Frey B. Mayer J. Mäder P. Widmer F. (2015). Distinct soil microbial diversity under long-term organic and conventional farming. *ISME J.* 9 1177–1194. 10.1038/ismej.2014.210 25350160 PMC4409162

[B30] Hou Q. Lin S. Ni Y. Yao L. Huang S. Zuo T. (2022). Assembly of functional microbial communities in paddy soil with long-term application of pig manure under rice-rape cropping system. *J. Environ. Manag.* 305:114374. 10.1016/j.jenvman.2021.114374 34953225

[B31] Ikoyi I. Egeter B. Chaves C. Ahmed M. Fowler A. Schmalenberger A. (2020). Responses of soil microbiota and nematodes to application of organic and inorganic fertilizers in grassland columns. *Biol. Fertil. Soils* 56 647–662. 10.1007/s00374-020-01440-5

[B32] Ingham E. R. (2009). “Chapter 4 - Soil biology primer: soil fungus,” in *Soil and Water Conservation* (Ankeny, IA: Soil & Water Conservation Society), 22–23.

[B33] Iqbal A. He L. Khan A. Wei S. Akhtar K. Ali I. (2019). Organic manure coupled with inorganic fertilizer: an approach for the sustainable production of rice by improving soil properties and nitrogen use efficiency. *Agronomy* 9:651. 10.3390/agronomy9100651

[B34] Jackson M. L. (1956). *). Soil Chemical Analysis—Advanced Course.* Madison, WI: University of Wisconsin, 991.

[B35] Jarvan M. Edesi L. Adamson A. Võsa T. (2014). Soil microbial communities and dehydrogenase activity depending on farming systems. *Plant Soil Environ.* 60 459–463. 10.17221/410/2014-PSE

[B36] Kaur G. Reddy M. S. (2014). Influence of P-solubilizing bacteria on crop yield and soil fertility at multilocational sites. *Eur. J. Soil Biol.* 61 35–40.

[B37] Klindworth A. Pruesse E. Schweer T. Peplies J. Quast C. Horn M. (2013). Evaluation of general 16S ribosomal RNA gene PCR primers for classical and next-generation sequencing-based diversity studies. *Nucleic Acids Res.* 41:e1. 10.1093/nar/gks808 22933715 PMC3592464

[B38] Kumar U. Shahid D. M. Tripathi R. Mohanty S. Kumar A. Bhattacharyya P. (2017). Variation of functional diversity of soil microbial community in sub-humid tropical rice-rice cropping system under long-term organic and inorganic fertilization. *Ecol. Indic.* 73 536–543.

[B39] Li P. Kong D. Zhang H. Xu L. Li C. Wu M. (2021). Different regulation of soil structure and resource chemistry under animal-and plant-derived organic fertilizers changed soil bacterial communities. *Appl. Soil Ecol.* 165:104020. 10.1016/j.apsoil.2021.104020

[B40] Li P. Liu M. Ma X. Wu M. Jiang C. Liu K. (2020). Responses of microbial communities to a gradient of pig manure amendment in red paddy soils. *Sci. Total Environ.* 705:135884. 10.1016/j.scitotenv.2019.135884 31818573

[B41] Li T. Zhang Y. Bei S. Li X. Reinsch S. Zhang H. (2020). Contrasting impacts of manure and inorganic fertilizer applications for nine years on soil organic carbon and its labile fractions in bulk soil and soil aggregates. *Catena* 194:104739. 10.1016/j.catena.2020.104739

[B42] Li R. Li M. Ashraf U. Liu S. Zhang J. (2006). Yield analysis of early indica rice Zhenguiai 1 in South China. *China Rice* 1:17.

[B43] Lima D. L. Santos S. M. Scherer H. W. Schneider R. J. Duarte A. C. Santos E. B. H. (2009). Effects of organic and inorganic amendments on soil organic matter properties. *Geoderma* 150 38–45. 10.1016/j.geoderma.2009.01.009

[B44] Liu J. Shu A. Song W. Shi W. Li M. Zhang W. (2021). Long-term organic fertilizer substitution increases rice yield by improving soil properties and regulating soil bacteria. *Geoderma* 404:115287. 10.1016/j.geoderma.2021.115287

[B45] Lu R. L. (2000). *Soil Agricultural Chemical Analysis Method.* Beijing: China Agricultural Science and Technology Press, 1–315.

[B46] Luo Y. Iqbal A. He L. Zhao Q. Wei S. Ali I. (2020). Long-term no-tillage and straw retention management enhances soil bacterial community diversity and soil properties in Southern China. *Agronomy* 10:1233. 10.3390/agronomy10091233

[B47] Merila P. Malmivaara-Lämsä M. Spetz P. Stark S. Vierikko K. Derome J. (2010). Soil organic matter quality as a link between microbial community structure and vegetation composition along a successional gradient in a boreal forest. *Appl. Soil Ecol.* 46 259–267. 10.1016/j.apsoil.2010.08.003

[B48] Ohyama T. Ito M. Kobayashi K. Araki S. Yasuyoshi S. Sasaki O. (1991). Analytical procedures of N, P, K contents in plant and manure materials using H2SO4-H2O2 Kjeldahl digestion method. *Bull. Fac. Agric. Niigata Univ.* 43 110–120.

[B49] Oladele S. O. Adeyemo A. J. Awodun M. A. (2019). Influence of rice husk biochar and inorganic fertilizer on soil nutrients availability and rain-fed rice yield in two contrasting soils. *Geoderma* 336 1–11. 10.1016/j.geoderma.2018.08.025

[B50] Pérez-Valera E. de Melo Rangel W. Elhottová D. (2022). Cattle manure application triggers short-term dominance of *Acinetobacter* in soil microbial communities. *Appl. Soil Ecol.* 176:104466. 10.1016/j.apsoil.2022.104466

[B51] Qaswar M. Jing H. Ahmed W. Li D. Liu S. Lu Z. (2020). Yield sustainability, soil organic carbon sequestration and nutrients balance under long-term combined application of manure and inorganic fertilizers in acidic paddy soil. *Soil Till. Res*. 198 1–6. 10.1016/j.still.2019.104569

[B52] Quast C. Pruesse E. Yilmaz P. Gerken J. Schweer T. Yarza P. (2012). The SILVA ribosomal RNA gene database project: improved data processing and web-based tools. *Nucleic Acids Res.* 41 D590–D596. 10.1093/nar/gks1219 23193283 PMC3531112

[B53] Robert C. P. Casella G. Casella G. (2010). *Introducing Monte Carlo Methods With R*, Vol. 18. New York, NY: Springer.

[B54] Rousk J. Bååth E. (2007). Fungal biomass production and turnover in soil estimated using the acetate-in-ergosterol technique. *Soil Biol. Biochem*. 39 2173–2177. 10.1016/j.soilbio.2007.03.023

[B55] Sapkota T. B. Singh L. K. Yadav A. K. Khatri-Chhetri A. Jat H. S. Sharma P. C. (2020). Identifying optimum rates of fertilizer nitrogen application to maximize economic return and minimize nitrous oxide emission from rice–wheat systems in the Indo-Gangetic Plains of India. *Arch. Agron. Soil Sci.* 66 2039–2054. 10.1080/03650340.2019.1708332

[B56] Sarwar G. Schmeisky H. Hussain N. Muhammad S. Ibrahim M. Safdar E. (2008). Improvement of soil physical and chemical improvement with compost application in rice-wheat cropping system. *Pak. J. Bot.* 40 275–282.

[B57] Schloss P. D. Westcott S. L. Ryabin T. Hall J. R. Hollister E. B. Hartmann M. (2009). Introducing mothur: open-source, platform-independent, community-supported software for describing and comparing microbial communities. *Appl. Environ. Microbiol.* 75 7537–7541. 10.1128/AEM.01541-09 19801464 PMC2786419

[B58] Schmidt S. Nemergut D. Darcy J. Lynch R. (2014). Do bacterial and fungal communities assemble differently during primary succession? *Mol. Ecol*. 23 254–258. 10.1111/mec.12589 26010467

[B59] Sharma G. D. Thakur R. Som R. Kauraw D. L. Kulhare P. S. (2013). Impact of integrated nutrient management on yield, nutrient uptake, protein content of wheat (T*riticum astivam*) and soil fertility in Typic Haplustert. *Bioscan* 8 1159–1160.

[B60] Stroobants A. Degrune F. Olivier C. Roisin C. Bodson B. Portetelle D. (2012). *Impact of Depth and Soil Compaction on Bacterial Diversity in Soil.* Copenhagen: University of Liège (ORBI).

[B61] Sundberg C. Al-Soud W. A. Larsson M. Alm E. Yekta S. S. Svensson B. H. (2013). 454 pyrosequencing analyses of bacterial and archaeal richness in 21 full-scale biogas digesters. *FEMS Microbiol. Ecol.* 85 612–626. 10.1111/1574-6941.12148 23678985

[B62] Tilahun-Tadesse F. Nigussie-Dechassa R. Wondimu B. Setegn G. (2013). Effect of farmyard manure and inorganic fertilizers on the growth, yield and moisture stress tolerance of rain-fed lowland rice. *Am. J. Res. Com.* 1 275–301.

[B63] Ullah S. Liang H. Ali I. Zhao Q. Iqbal A. Wei S. (2020). Biochar coupled with contrasting nitrogen sources mediated changes in carbon and nitrogen pools, microbial and enzymatic activity in paddy soil. *J. Saudi Chem. Soc.* 24 835–849. 10.1016/j.jscs.2020.08.008

[B64] van Bruggen A. H. C. Sharma K. Kaku E. Karfopoulos S. Zelenev V. V. Blok W. J. (2015). Soil health indicators and Fusarium wilt suppression in organically and conventionally managed greenhouse soils. *Appl. Soil Ecol*. 86 192–201. 10.1016/j.apsoil.2014.10.014

[B65] van der Heijden M. G. Bardgett R. D. van Straalen N. M. (2008). The unseen majority: soil microbes as drivers of plant diversity and productivity in terrestrial ecosystems. *Ecol. Lett.* 11 296–310. 10.1111/j.1461-0248.2007.01139.x 18047587

[B66] Vance E. D. Brookes P. C. Jenkinson D. S. (1987). Microbial biomass measurements in forest soils: the use of the chloroform fumigation-incubation method in strongly acid soils. *Soil Biol. Biochem.* 19 697–702. 10.1016/0038-0717(87)90051-4

[B67] Verma P. Yadav A. N. Kumar V. Singh D. P. Saxena A. K. (2017). “Beneficial plant-microbes interactions: biodiversity of microbes from diverse extreme environments and its impact for crop improvement,” in *Plant-Microbe Interactions in Agro-Ecological Perspectives*, eds Singh D. Singh H. Prabha R. (Singapore: Springer), 543–580.

[B68] Wagg C. Schlaeppi K. Banerjee S. Kuramae E. E. van der Heijden M. G. A. (2019). Fungal-bacterial diversity and microbiome complexity predict ecosystem functioning. *Nat. Commun.* 10:4841. 10.1038/s41467-019-12798-y 31649246 PMC6813331

[B69] Wang J. L. Liu K. L. Zhao X. Q. Zhang H. Q. Li D. Li J. J. (2021). Balanced fertilization over four decades has sustained soil microbial communities and improved soil fertility and rice productivity in red paddy soil. *Sci. Total Environ.* 793:148664. 10.1016/j.scitotenv.2021.148664 34328991

[B70] Wang S. Tian H. Liu J. Pan S. (2003). Pattern and change of soil organic carbon storage in China: 1960s–1980s. *Tellus B* 55 416–427. 10.1034/j.1600-0889.2003.00039.x 11841302

[B71] Wei M. Hu G. Wang H. Bai E. Lou Y. Zhang A. (2017). 35 years of manure and chemical fertilizer application alters soil microbial community composition in a Fluvo-aquic soil in Northern China. *Eur. J. Soil Biol.* 82 27–34. 10.1016/j.ejsobi.2017.08.002

[B72] Wu Z. X. Li H. H. Liu Q. L. Changyan Y. Faxin Y. (2020). Application of bio-organic fertilizer, not biochar, in degraded red soil improves soil nutrients and plant growth. *Rhizosphere* 16:100264. 10.1016/j.rhisph.2020.100264

[B73] Xu X. Thornton P. E. Post W. M. (2013). A global analysis of soil microbial biomass carbon, nitrogen and phosphorus in terrestrial ecosystems. *Glob. Ecol. Biogeogr.* 22 737–749. 10.1111/geb.12029

[B74] Yang X. D. Ni K. Shi Y. Z. Yi X. Y. Zhang Q. F. Fang L. (2018). Effects of long-term nitrogen application on soil acidification and solution chemistry of a tea plantation in China. *Agric. Ecosyst. Environ* 252 74–82. 10.1016/j.agee.2017.10.004

[B75] Yu Y. Wu M. Petropoulos E. Zhang J. Nie J. Liao Y. (2019). Responses of paddy soil bacterial community assembly to different long-term fertilizations in southeast China. *Sci. Total Environ*. 656 625–633. 10.1016/j.scitotenv.2018.11.359 30529966

[B76] Zhang J. Kobert K. Flouri T. Stamatakis A. (2014). PEAR: a fast and accurate illumina paired-end read merger. *Bioinformatics* 30 614–620. 10.1093/bioinformatics/btt593 24142950 PMC3933873

[B77] Zhang M. J. Jia J. Q. Hua L. U. Feng M. C. Yang W. D. (2021). Functional diversity of soil microbial communities in response to supplementing 50% of the mineral N fertilizer with organic fertilizer in an oat field. *J. Integr. Agric.* 20 2255–2264. 10.1016/S2095-3119(20)63331-7

[B78] Zhang Y. L. Sun C. X. Chen Z. H. Zhang G. N. Cheng L. J. Wu Z. J. (2019). Stoichiometric analyses of soil nutrients and enzymes in a Cambisol soil treated with inorganic fertilizers or manures for 26 years. *Geoderma* 353 382–390. 10.1016/j.geoderma.2019.06.026

[B79] Zhao J. Zhang R. Xue C. Xun W. Sun L. Xu Y. (2014). Pyrosequencing reveals contrasting soil bacterial diversity and community structure of two main winter wheat cropping systems in China. *Microb. Ecol.* 67 443–453. 10.1007/s00248-013-0322-0 24276539

[B80] Zhu J. Peng H. Ji X. Li C. Li S. (2019). Effects of reduced inorganic fertilization and rice straw recovery on soil enzyme activities and bacterial community in double-rice paddy soils. *Eur. J. Soil Biol.* 94:103116. 10.1016/j.ejsobi.2019.103116

