# Correction: Partial substitution of organic fertilizer with chemical fertilizer improves soil biochemical attributes, rice yields and restores bacterial community diversity in a paddy field

**DOI:** 10.3389/fpls.2025.1691367

**Published:** 2025-09-11

**Authors:** Anas Iqbal, Liang He, Izhar Ali, Pengli Yuan, Abdullah Khan, Zhang Hua, Shanqing Wei, Ligeng Jiang

**Affiliations:** ^1^ College of Life Science and Technology, Guangxi University, Nanning, China; ^2^ College of Agriculture, Guangxi University, Nanning, China

**Keywords:** paddy field, organic manure, chemical fertilizer, soil chemical properties, bacterial community, grain yield

The title of this article was erroneously given as: “Partial Substation of Organic Fertilizer with Chemical Fertilizer Improves Soil Biochemical At**t**ributes, Rice Yields and Restores Bacterial Community Diversity in a Paddy Field”. The correct title of the article is “Partial Substitution of Organic Fertilizer with Chemical Fertilizer Improves Soil Biochemical Attributes, Rice Yields and Restores Bacterial Community Diversity in a Paddy Field”.

There is also an error in the **Keywords**. The word “soil chemical peripeties” has been corrected to “soil chemical properties”.

The original version of this article has been updated.

